# Evaluation of surgical treatment for incidental gallbladder carcinoma diagnosed during or after laparoscopic cholecystectomy: single center results

**DOI:** 10.1186/s13104-017-2387-1

**Published:** 2017-01-21

**Authors:** Masashi Utsumi, Hideki Aoki, Tomoyoshi Kunitomo, Yutaka Mushiake, Isao Yasuhara, Takashi Arata, Koh Katsuda, Kohji Tanakaya, Hitoshi Takeuchi

**Affiliations:** Department of Surgery, National Hospital Organization, Iwakuni Clinical Center, 1-1-1 Atago-machi, Iwakuni, Yamaguchi 740-8510 Japan

**Keywords:** Incidental gallbladder carcinoma, Laparoscopic cholecystectomy, Surgical treatment

## Abstract

**Background:**

Laparoscopic cholecystectomy (LC) is the accepted standard management for benign gallbladder disease. LC rarely results in a diagnosis of incidental gallbladder carcinoma (IGBC). The aim of our study was to report our experience with IGBC diagnosed during or following LC.

**Methods:**

Between January 2008 and January 2015, 352 patients underwent LC at Iwakuni Clinical Center. Among these patients, 8 (2.3%) were diagnosed with IGBC. We evaluated their characteristics, surgical related variables, histopathological findings and surgical outcomes.

**Results:**

Patient median age was 71 (range 49–88) years, and 3 out of 8 were female. All patients with IGBC were Japanese. The grade of cancer was as follows: pT1a (3 cases), pT2 (4 cases) and pT3 (1 case). Two patients with pT2 disease underwent radical surgery. The median follow-up time of these patients was 24 (range 11–80) months. All patients are still alive and two of three patients who refused radical surgery have developed recurrence (liver metastases and recurrence in the peritoneum).

**Conclusions:**

Although the number of cases was small, the results of this study further support the suggestion that gallbladder carcinoma may be curable if diagnosed as IGBC at an early stage. If the cancer has reached an advanced stage, radical surgery should be performed.

## Background

Gallbladder cancer (GBC) is a relatively rare neoplasm and is considered to be an aggressive and highly lethal disease. It is the most frequently occurring malignancy of the biliary tract and the fifth most common gastrointestinal cancer [1]. Only 30% of gallbladder carcinomas are suspected preoperatively, and the remaining 70% are usually discovered incidentally by pathological examination during or after surgery [2].

In recent years, laparoscopic cholecystectomy (LC) has become the accepted gold standard management for gallbladder disease. With the advantages of a shorter hospital stay, decreased post-operative pain and earlier resumption of normal activities compared to open cholecystectomy, this procedure has now become routine in the treatment of benign gallbladder disease worldwide. LC that is performed for benign gallbladder disease rarely results in a diagnosis of unexpected gallbladder carcinoma. Incidental gallbladder carcinoma (IGBC) is defined as carcinoma of the gallbladder suspected for the first time during cholecystectomy, or accidentally found on histological examination of gallbladder. The estimated incidence of IGBC diagnosed by cholecystectomy is 1–2% [3]. Although patients with GBC have a poor prognosis, most IGBC tumors tend to be at an early stage of development. Hence, radical second surgery is performed in most patients with IGBC, and improved prognosis is usually reported [4–7]. However management of IGBC is a difficult issue in the absence of established guidelines. The primary aim of our study was to evaluate the incidence, clinicopathological characteristics and outcome of patients with IGBC.

## Methods

The study was approved by Ethics Committee at Iwakuni Clinical Center. Between January 2008 and January 2015, 352 patients underwent LC at Iwakuni Clinical Center. Among these patients, 8 (2.3%) were diagnosed with IGBC. Their clinical presentation, ultrasonography findings, preoperative diagnosis, intraoperative findings, histopathological records and surgical outcomes were reviewed. We excluded from the study all patients with suspected pre-operative malignancy. All LCs were executed using the standard 4 trocar technique, and EndBag protected gallbladder (GB) extraction. Patients were staged according to the American Joint Committee on Cancer 7th edition tumor node metastasis clinical staging system for GBC [8]. We have advocated additional radical surgery for patients with pT2 or pT3 tumors [4, 5]. Regarding the surgical radicalization procedure, we performed hepatic S4a + S5 resection with hepatic peduncle lymphadenectomy [4, 5, 9]. The completeness of the resection was classified as follows: R0, no residuals in the hepatic margins; R1, a microscopically positive margin; and R2 macroscopic residuals in the hepatic margin.

## Results

The clinical characteristics of patients with IGBC during or following LC are detailed in Table 1. Of the eight patients three were female, and the median age of all patients was 71 (range, 49–88) years. All patients with IGBC were Japanese. Five patients presented with complaints of pain in the right hypochondrium. Ultrasonography revealed a thickened gallbladder in four patients and multiple polyps in two. Gall stones were observed in five patients. No patient exhibited pancreaticobiliary malfunction on magnetic resonance cholangiopancreatography. The preoperative diagnosis of the eight patients with incidental IGBC was as follows: cholecystitis (2 cases), gallbladder polyps (2 cases), gallbladder stones (4 cases) and adenomyomatosis (2 cases). There was some overlapping.

**Table 1:** Clinical characteristics of patients with incidental gallbladder carcinoma during or following laparoscopic cholecystectomy

Patient number	Clinical presentation	Pre-ALP (units/L)	Ultrasonographic finding	Preoperative diagnosis
1	None	214	Multiple GB polyps	GB polyp
2	Right hypochondralgia	315	Multiple GB stones	GB stone
3	None	178	Multiple GB polyps	GB polyp
4	Right hypochondralgia	365	Multiple GB stones	GB stone, cholecystitis
5	Right hypochondralgia	272	GB wall thickening, GB stones	GB stone, cholecystitis
6	None	189	Segmental GB wall thickening	Adenomyomatosis
7	Right hypochondralgia	132	Segmental GB wall thickening, multiple GB stones	Adenomyomatosis
8	Right hypochondralgia	313	Segmental GB wall thickening, multiple GB stones	GB stone

*GB* gallblader, *Pre*-*ALP* preoperative serum level of alkaline phosphatase

The histopathological characteristics and surgical outcomes of IGBC patients during or following LC are detailed in Table 2. Intraoperatively, in one patient, there was a tumor suspected of being an adenocarcinoma, and frozen sectioning was performed; a diagnosis of adenocarcinoma was confirmed. The GBC was found to have invaded the transverse colon. Consequently, we abandoned open cholecystectomy with removal of cystic duct lymph node and performed colectomy. Also, in case with swelling cystic duct node, we removed it. The remaining seven patients were diagnosed postoperatively on the basis of the histopathological examination.

**Table 2:** Histopathological characteristics and surgical outcome of patients with incidental gallbladder carcinoma during or following laparoscopic cholecystectomy

Patient number	Diagnosis	Removal of cystic duct node in 1st operation	Hospital stay (days)	Postoperative complication	Tumor location of gallbladder	Size (cm)	Pathology	pT	2nd surgical procedure	N	M	Stage	Margin	Recurrence	Outcome (months)
1	Postoperatively	None	6	None	Body	0.8	Well differentiated adenocarcinoma	1a					R0	None	Alive (80)
2	Postoperatively	None	5	None	Fundus-body	4.5	Well differentiated adenocarcinoma	2	Radicalization	0	0	II	R0	None	Alive (51)
3	Postoperatively	None	6	None	Body	1.7	Well differentiated adenocarcinoma	1a					R0	None	Alive (40)
4	Postoperatively	Yes	5	None	Neck	3.3	Moderately differentiated adenocarcinoma	2	Refused	1	0	IIIB	R1	Liver	Alive (28)
5	Intraoperatively	Yes	10	None	Fundus	2	Poorly differentiated adenocarcinoma	3	Refused	1	0	IIIB	R2	Peritoneum	Alive (24)
6	Postoperatively	None	9	None	Body	2.3	Well differentiated adenocarcinoma	2	Radicalization	0	0	II	R0	None	Alive (22)
7	Postoperatively	None	5	None	Fundus-body	6.5	Well differentiated adenocarcinoma	1a					R0	None	Alive (21)
8	Postoperatively	None	13	SSI	Fundus-body	6	Well differentiated adenocarcinoma	2	Refused	0	0	II	R1	None	Alive (11)

*pT* pathological T stage according to AJCC 6th edition, *SSI* surgical site infection

According to the pathological TNM classification system, the tumor stages were as follows: pT1a (3 cases); pT2 (4 cases); and pT3 (1 case). The pathological characteristics of the tumors were as follows: well differentiated adenocarcinoma (6 cases); moderately differentiated adenocarcinoma (1 case); and poorly differentiated adenocarcinoma (1 case).

Three patients with pT1a disease had no additional surgery, and there was no recurrence. Two patients with pT2 disease underwent radical surgery and there was no recurrence. The remaining three patients with pT2 disease (2 cases) or pT3 disease (1 case) refused radical surgery. All patient had no adjuvant therapy (chemotherapy or chemoradiation). Follow up of both patients undergoing radical surgery and refusing radical surgery is blood examination including tumor marker every 2 months and CT imaging studies every 4 months. The median follow-up time was 24 (range 11–80) months. All patients are still alive and two of the three patients who refused radical surgery developed recurrence (liver metastases and recurrence in the peritoneum, respectively). The remaining patients were disease-free during follow-up. We did not find any patients with port-site recurrence during the follow-up period. Patients with pT2/T3 received no adjuvant chemotherapy or chemoradiation post-operatively.

## Discussion

The incidence of IGBC during or after LC has been reported to be 0.19–3.3% [5, 10–12]. Because of the increased use of LC and difficulty regarding the preoperative diagnosis of GBC, IGBC has become more frequent [11, 13]. In the present study, the rate of occurrence of IGBC was 2.3%.

Some studies have reported that risk factors for IGBC are as follows: sex (female); obesity; age >65 years; cholelithiasis; polypoid lesions; Asian or African American; and an elevated alkaline phosphatase level [14–17]. In our study, three patients were female and their median age was 64 years. All of our patients with IGBC were Asian. The symptoms related to IGBC can be relatively nonspecific, with the early symptoms mimicking those of GB stones or cholecystitis. GB stones are found in 70–98% of patients with GBC. Cholelithiasis causes chronic irritation and inflammation of the GB, which leads to the development of mucosal dysplasia and subsequent carcinoma; it takes a long time for the promotion of tumor proliferation and the occurrence of malignancy [18]. In our study, pain in the right hypochondrium was present in four patients. An additional three patient had no symptoms. None of our patients presented with a clinical profile that differed from that of typical patients with GB stones or cholecystitis. In the early stages, it is difficult to differentiate between GBC and cholecystitis, because thickening of the GB wall is a feature of both diseases [19].

Frozen sectioning should be performed when surgeons suspect malignancy in intraoperative finding [20, 21]. In our study, the intraoperative assessment of the GB using frozen sections revealed GBC in one patient with GB wall thickening and cholecystitis. Consequently, we switched from LC to open cholecystectomy. The efficacy of frozen sections was poor with respect to the diagnosis of carcinoma in situ [20, 21]. Kwon et al. also found that diagnosis using frozen sections did not reliably detect the carcinoma in situ and the depth of invasion of GBC; thus, it should not be considered a definitive diagnostic procedure [22]. The most appropriate approach for diagnosis at present is to macroscopically examine the GB mucosa during surgery, and to perform frozen sectioning for any suspected lesion.

The survival rate of IGBC patients is high with a median survival rate of 21.2–60 months [5, 10, 11]. All patients in our studies were alive at a mean follow-up time of 24 months. One reason for this may be early diagnosis, as in the present study in which most of the cases involved stage 1 or 2 tumors, thereby resulting in better prognoses. However, we could not find any common characteristic that could differentiate these GB carcinomas from benign tumors in laboratory and sonographic findings. The difficulty in early diagnosis of GBC results from its poor specificity and ambiguity regarding clinical symptoms [23–25]. Tumor differentiation is an independent prognostic factor that affects survival [5]. In the present study, six of eight tumors were well differentiated adenocarcinomas.

Surgical resection is the only curative therapy for GBC [15, 26, 27]. Determining the therapeutic approach for GBC in accordance with disease stage is supported by most authors [27, 28]. There is a consensus that simple cholecystectomy or LC is an adequate treatment for pT1a. pT2 and higher stages should be treated using additional radical surgery using hepatic S4a + S5 resection with hepatic peduncle lymphadenectomy [9, 15, 29, 30]. For patients with T1b disease, the correct approach is still debated and some authors recommend hepatic gallbladder bed resection with hepatic peduncle lymphadenectomy [31, 32]. In the present study, the survival of patients with pT1 disease was favorable after LC. Two patients with pT2 disease underwent additional radical surgery and had good outcomes. Other patients with pT2 and pT3 disease refused additional radical surgery and later developed recurrence.

Laparoscopic cholecystectomy appears to have resulted in the earlier discovery of GBC in some patients, resulting in an increased probability of successful treatment [33]. Precise surgery is essential in obtaining a good outcome from IGBC treatment [4]. The present study was retrospective and involved a small number of patients. A study involving a considerably larger sample size will be required to evaluate the surgical outcome and effectiveness of radical surgery for IGBC.

## Conclusions

The incidence of IGBC detected during laparoscopic cholecystectomy in this single center study was found to be 2.3%. No association could be found with risk factors that have been reported by other authors. Although the number of patients enrolled was small, our findings further support the suggestion that GB carcinoma may be curable if diagnosed at an early stage as IGBC. pT1b or greater tumor stages identified on LC may benefit from additional radical resection.

## Abbreviations

GB: gallbladder
GBC: gallbladder cancer
IGBC: incidental gallbladder carcinoma
LC: laparoscopic cholecystectomy
